# Dermal Toxicity Influence of Gold Nanomaterials after Embedment in Cosmetics

**DOI:** 10.3390/toxics10060276

**Published:** 2022-05-24

**Authors:** Chusheng Liu, Yanjing Wang, Gaofei Zhang, Xuebin Pang, Jiao Yan, Xiaoou Wu, Yingheng Qiu, Ping Wang, Houshuang Huang, Xiaowei Wang, Haiyuan Zhang

**Affiliations:** 1 NMPA Key Laboratory for Monitoring and Evaluation of Cosmetics, Shenzhen Institute for Drug Control, Shenzhen 518057, China; chusheng_liu@foxmail.com (C.L.); ahlqzgf@163.com (G.Z.); pangxueb@sina.com (X.P.); xiaoouwu@szidc.org.cn (X.W.); qiuyingheng@szidc.org.cn (Y.Q.); wangping662@sina.com (P.W.); huanghsh@szidc.org.cn (H.H.); 2 Changchun Institute of Applied Chemistry, Chinese Academy of Sciences, Changchun 130022, China; yanjwang@ciac.ac.cn (Y.W.); jiaoyan@ciac.ac.cn (J.Y.)

**Keywords:** gold nanomaterials, cosmetics, toxicity reduction, skin

## Abstract

Gold nanomaterials (Au NMs) have been widely used in cosmetic products for improving the brightening, and reducing the wrinkling of, skin, etc.; however, the dermal safety of Au NMs is rarely concerned. A previous study found that cosmetics could enhance the toxicity of Au nanosheets, but different physicochemical properties of Au NMs will induce different interaction modes with ingredients of cosmetics, potentially leading to different toxicity profiles. In the present study, spherical and rodlike Au NMs were first found in commercial cosmetics, and then Au nanospheres (NSs) with different sizes and Au nanorods (NRs) with different aspect ratios were prepared to simulate these Au NMs in cosmetics and further investigate their toxicity before and after embedment in cosmetics. It was found that the primary sizes, morphologies, and optical absorptions of these Au NSs and NRs before and after embedment were similar; however, their hydrodynamic sizes and zeta potentials were noticeably different. Then, these Au NSs and NRs presented weak or no cytotoxicity against HaCaT keratinocytes, while cosmetic cream could alleviate their cytotoxicity. Moreover, the cream could enhance the accumulation of Au NSs and NRs in the skin of hairless mice, but it also alleviated the toxicological responses of Au NSs and NRs in terms of superoxide dismutase (SOD) elevation and malondialdehyde (MDA) reduction. Therefore, the embedment of Au NSs and NRs into cosmetics can alleviate the in vitro and in vivo dermal toxicities of Au NSs and NRs.

## 1. Introduction

The global cosmetic industry has rapidly developed, showing ~4.5% of average annual growth in the past twenty years. The rapid rise of the cosmetic industry drives a series of innovative products constantly designed on the basis of increasingly detailed scientific knowledge [1]. The advent of nanotechnology has made a huge contribution to the progress in the cosmetic industry, and many cosmetic products have been developed using nanotechnology, leading to a major breakthrough in innovative product generation. The superior properties of nanomaterials overcome the common limitations of cosmetics by enhancing penetration, improving the stability of ingredients, controlling the release of active ingredients, and functioning themselves as active agents [2]. Nanomaterials have been extensively applied in sunscreen creams [3,4], anti-aging creams [5,6], hair products [7,8], facial masks [9,10], and also in lipsticks [11]. It indicates that nanomaterials are gaining an increasing interest in cosmetic manufacturing. Despite the huge potential benefits of nanomaterials, concerns have been raised about their safety aspects. The European Commission (EC) has updated the guidance on the safety assessment of nanomaterials (NMs) in cosmetics [12], and the United States (US) Food and Drug Administration (FDA) has developed its guidance for the application of nanotechnology in industrial products [13]. Since NMs contained in cosmetics potentially can enter the body mainly through the route of dermal absorption, the complicated physicochemical properties of NMs, such as composition, size, shape, charge, dissolution, aggregation, crystallographic facet, and surface reactivity, etc., can trigger various toxicological responses and potentially cause adverse health effects, which has been demonstrated by the Scientific Committee on Consumer Products (SCCP) [14]. Evaluating the safety of nanocosmetics has become an important issue not only for customers but also for manufacturers [15].

Gold nanomaterials (Au NMs) have been widely used in cosmetic products, capable of reducing the wrinkling of skin [16], improving skin brightening, promoting skin healing, having a cleansing effect, reducing inflammation and reactive oxygen species damage, and slowing down the collagen depletion [17] and elastin breakdown [18]. The ease of synthesis and surface functionalization as well as the inherent biocompatibility of Au NMs have significantly contributed to these applications. At the same time, the safety of Au NMs on the skin has been of concern. A size-dependent permeation through rat skin was found on 15, 102, and 198 nm Au nanospheres (NSs) synthesized through citrate reduction methods, where 15 nm Au NSs showed a higher penetration than 102 and 198 nm Au NS [19]. The influence of the surface charge of Au nanorods (NRs) on skin penetration was also investigated using a Franz-type diffusion cell (FDC). Negatively charged Au NRs showed deeper penetration than positively charged Au NRs on rat skin. In addition, it has also been clarified that Au NSs are inherently safe to skin cells, while Au NRs are toxic due to the coating molecules (e.g., cetyltrimethylammonium bromide (CTAB)). Although these studies have revealed the basic penetration and toxicity behaviors of Au NMs on skin cells and tissues, most of them only focus on raw Au NMs. Considering that the complicated ingredients of cosmetics potentially may alter the physicochemical properties of Au nanomaterials, it will be very necessary to understand the toxicity behaviors of Au NMs embedded in cosmetics. 

Recently, pilot studies have reported to successfully separate Au nanosheets from commercial cosmetics and investigate their toxicity on human keratinocytes and guinea pig skin [20,21]. It was found that cosmetic cream enhanced the cytotoxicity of Au nanosheets in HaCaT keratinocytes, which is ascribed to the toxic ingredients of cosmetic cream. Since Au nanosheets are two-dimensional NMs, the significantly different physicochemical properties and toxicity profiles from zero-dimensional NMs are usually exhibited. Based on our preliminary study, various nanoscale spheres and rods were also observed in commercial gold cosmetics products; however, on the one hand, the amount of separated Au NMs was insufficient for toxicity evaluation, and, on the other hand, it was hard to further purify the individual NMs (NSs or NRs) for clarification of their toxicity profiles. Therefore, in the present study, we synthesized different sized Au NSs and NRs and completely embedded them in cosmetic cream. After extraction, the physicochemical properties of extracted Au NSs and NRs were compared with those of raw ones, and the cellular and dermal toxicities of these Au NMs and the Au NM-embedded cream were assessed in keratinocytes (HaCaT) and the hairless mice.

## 2. Material and Methods

### 2.1. Materials

Gold chloride trihydrate (HAuCl_4_·3H_2_O, 99%), sodium citrate, and cetyltrimethylammonium bromide (CTAB) were obtained from Sigma-Aldrich. Nitric acid (HNO_3_, 65%), hydrogen peroxide (H_2_O_2_, 30%), hydrochloric acid (HCl, 37%), hexane (>98%), sodium dodecyl sulfate (SDS, >98%), and 50 nm zinc oxide (ZnO) nanoparticles were all purchased from Aladdin Chemicals (Shanghai, China). All reagents were used without further purification. High purity deionized water produced by Milli-Q plus system (Merck, Darmstadt, Germany) was used for all experiments.

### 2.2. Extraction of Au NMs from Cream

The Au NMs were extracted from commercial facial cream, according to a previously established method with slight modifications [20]. Briefly, 1.0 g of commercial facial cream containing Au NMs was transferred into a 50-mL centrifuge tube, and 10 mL of water was added, followed by vigorous vortexing to disperse the cream. Then, 10 mL of hexane was added and vortexed for 15 s. The suspension was centrifuged for 15 min at 12,000 rpm after 15 min of ultrasonication. The supernatant (hexane layer) was then carefully removed, and the precipitate was re-dispersed in 20 mL of H_2_O/ethanol (1/1, *v*/*v*). Following 15 s of vigorous vortexing and 10 min of ultrasonication, the solution was filtered through a 0.22-μm PVDF membrane (Millipore, Burlington, MA, USA). Then, the filtrate was centrifuged at 12,000 rpm for 10 min, and the precipitate was re-dispersed in 2 mL of deionized (DI) water for further analysis.

### 2.3. Physicochemical Characterization 

Ultraviolet-visible (UV-Vis) absorption spectra were collected using a UV2450 spectrophotometer (Shimadzu, Kyoto, Japan). Dynamic light scattering (DLS) and zeta potential were measured using the Zetasizer Lab Blue instrument (Malvern Panalytical, Malvern, Worcs, UK). Transmission electron microscopy (TEM) imaging was performed in a Hitachi HT7800 TEM system at 100 kV. Elemental content was determined by iCAP RQ inductively coupled plasma mass spectrometry (ICP-MS) (Thermo, Waltham, MA, USA). For ICP-MS measurements in single-particle mode (spICP-MS), experiments were conducted on a NexION 350X ICP-MS instrument (PerkinElmer, Waltham, MA, USA), which was equipped with Syngistix Nano application software. 

### 2.4. Synthesis of Au NS1-3

Au seeds were synthesized through a citrate-reduced method [22]. Briefly, 2.2 mmol L^−1^ sodium citrate solution was heated until boiling for 15 min under vigorous stirring. Then, 1 mL of HAuCl_4_ (25 mmol L^−1^) was injected and stirred for 10 min. After cooling to 90 °C, 1 mL of sodium citrate (60 mmol L^−1^) and 1 mL of HAuCl_4_ (25 mmol L^−1^) were sequentially injected into the solution and stirred for 30 min. To obtain Au NS1, Au NS2, and Au NS3, this step could be repeated 3, 9, and 11 times, respectively, followed by 15 min of centrifugation at 8000 rpm to remove the supernatant.

### 2.5. Synthesis of Au NR1-3

Au NRs were prepared by the seed growth method, according to a previous study with modification [23].

Preparation of Au seed solution: 0.1 mL of HAuCl_4_ (25 mmol L^−1^) was added into 10 mL of CTAB (0.1 mol L^−1^) solution, and 600 μL of ice-cold sodium borohydride (NaBH_4_) (10 mmol L^−1^) was added into the above mixture with vigorous stirring. The achieved solution was aged for 3 h at 30 °C.

*Synthesis of CTAB-stabilized Au NR1*: 100 mL of CTAB (0.1 mol L^−1^) was mixed with 2 mL of HAuCl_4_ (25 mmol L^−1^), 45 μL of silver nitrate (AgNO_3_) (0.1 mol L^−1^), 1 mL of sulfuric acid (H_2_SO_4_) (1 mol L^−1^), and 100 μL of cupric chloride (CuCl_2_) (0.1 mol L^−1^) to form a homogenous solution. Then, 800 μL of ascorbic acid (AA) (0.1 mol L^−1^) was added to the solution, followed by the addition of 240 μL of the above seed solution. The resulting mixture was kept constant for 12 h at 30 °C. Finally, 1.14 mL of HAuCl_4_ (25 mmol L^−1^) was added and kept constant for 2 h. The suspension was centrifuged at 8000 rpm for 10 min to achieve CTAB-stabilized Au NR1 as precipitates and further dispersed in 30 mL of deionized (DI) water. 

*Synthesis ofCTAB-stabilized Au NR2*: 100 mL of CTAB (0.1 mol L^−1^) was mixed with 2 mL of HAuCl_4_ (25 mmol L^−1^) and 120 μL of AgNO_3_ (0.1 mol L^−1^) to form a homogenous solution. Then, 552 μL of AA (0.1 mol L^−1^) was added to the solution, followed by an addition of 120 μL of the above seed solution. The resulting mixture was kept constant for 12 h at 30 °C. Finally, 55.2 μL of AA was added and kept for 40 min, and this step was repeated one time. The suspension was centrifuged at 8000 rpm for 10 min to achieve CTAB-stabilized Au NR2 as precipitates and further dispersed in 30 mL of DI water.

*Synthesis ofCTAB-stabilized Au NR3*: 100 mL of CTAB (0.1 mol L^−1^) was mixed with 2 mL of HAuCl_4_ (25 mmol L^−1^), 110 μL of AgNO_3_ (0.1 mol L^−1^), and 1 mL of H_2_SO_4_ (1 mol L^−1^) to form a homogenous solution. Then, 800 μL of AA (0.1 mol L^−1^) was added to the solution, followed by an addition of 1 mL of the above seed solution. The resulting mixture was kept constant for 1 h at 30 °C. Finally, 1 mL of HAuCl_4_ (25 mmol L^−1^) and 400 μL AA (0.1 mol L^−1^) were added and kept for 2 h. The suspension was centrifuged at 8000 rpm for 10 min to achieve CTAB-stabilized Au NR3 as precipitates and further dispersed in 30 mL of DI water. 

*Synthesis of Au NR1-3*: 20 mL of CTAB-stabilized Au NR1-3 was re-dispersed in 100 mL of 0.15 wt.% polystyrene sulfonate (PSS) solution, making the optical density (O.D.) at 400 nm reach 1. The obtained suspension was stirred for 1 h and then centrifuged at 8000 rpm for 10 min. This step was repeated 2 times and the final precipitates as PSS-stabilized Au NRs were re-dispersed into 20 mL of 0.7 wt.% PSS. Then, 2 mL of obtained PSS-stabilized Au NR1-3 was centrifuged at 8000 rpm for 10 min to remove redundant PSS surfactants and re-dispersed in 30 mL of 5 mmol L^−1^ sodium citrate (O.D.~1), followed by stirring for 12 h. Then, the suspension was centrifuged at 8000 rpm for 10 min and washed with 5 mmol L^−1^ sodium citrate 3 times to achieve citrate-stabilized Au NRs as precipitates.

### 2.6. Preparation of Au NM-Embedded Cream (Au NM-Cream) and Extraction of Au NMs

The same commercial facial cream, but without the Au NM ingredient (confirmed by ICP-MS), was chosen as the blank cream. Then, 50 μL of various Au NSs or NR suspension (0.37 mg/mL) was fully mixed with 10 g of cream, and the resulting Au NM-Cream was stirred overnight at 40 °C. Then, this Au NS- or NR-embedded cream (Au NS-Cream or Au NR-Cream) was kept at room temperature for 24 h. The homogeneity of Au in the cream was assessed by determining the Au content of three random aliquots using ICP-MS analysis and expressed as the relative standard deviation (RSD%). After complete suspension, Au NSs or NRs were extracted from the Au NS-cream or Au NR-Cream, according to the steps described in Section 2.2. The achieved Au NS or NRs were denoted as extracted Au NSs or NRs. 

### 2.7. Cell Culture 

Human keratinocyte (HaCaT) cells were cultured in a vented 75-cm^2^ flask (Corning, Fisher Scientific) loaded with Dulbecco’s Modified Eagle’s Medium (DMEM), containing 4.5 g L^−1^ D-glucose, 110 mg L^−1^ sodium pyruvate and L-glutamine (Gibco), 10% fetal calf serum (Gibco), 100 U mL^−1^ penicillin, and 100 μg mL^−1^ streptomycin. A Forma Steri-Cycle i250 CO_2_ incubator (Thermo Scientific) conditioned with 5% CO_2_ and a humidified environment at 37 °C was employed for cell incubation.

### 2.8. Cell Viability Assessment 

The viability of HaCaT cells exposed to Au NMs or Au NM-Cream at various surface area dosages (equivalent to Au content) was measured by the CCK-8 assay kit (Beyotime, Shanghai, China). Considering that the daily usages of facial cream and face area are 2 g and 600 cm^2^ [21,24], respectively, the daily surface area dosage of facial cream was calculated to be 3.33 mg cm^−2^. Based on the Au content (1.85 mg kg^−1^ cream) in facial cream, the daily surface area dosage was 6.1 ng cm^−2^. To be consistent with 30 days of in vivo treatment, the cells were treated for 24 h, but the highest dosage of Au NMs was set at 0.18 μg cm^−2^. Typically, 1 × 10^4^ HaCaT cells in 100 μL of medium were seeded in each well of 96-well plates (Corning, Fisher Scientific) for overnight growth. Then, the medium was removed, and the cells were washed with phosphate buffered saline (PBS) three times and further incubated with 100 μL of fresh DMEM containing various concentrations (0–0.57 μg mL^−1^) of Au NMs or Au-Cream (equivalent to Au content) for 24 h. ZnO nanoparticles in the range of 0–200 μg mL^−1^ were used as the positive control. Then, 10 μL of CCK-8 was added to each well, followed by another 2 h incubation. The plates were directly measured by a microplate reader (Varioskan Flash, Thermo Fisher Scientific, Vantaa, Finland) at 450 and 650 nm of wavelength. 

Cell viability was also assessed through live/dead cell staining [25]. After 24 h of exposure to 0.18 μg cm^−2^ Au NSs or NRs (equivalent to Au content), HaCaT cells were washed with PBS three times and stained with 1 μmol L^−1^ calcein acetoxymethyl ester (Calcein AM) (Beyotime, Shanghai, China) and 1 μmol L^−1^ propidium iodide (PI) (Beyotime, Shanghai, China) for 15 min. The fluorescence imaging was acquired by an Axio Observer 7 system (ZEISS, Oberkochen, Germany).

*Cellular glutathione (GSH) measurements:* GSH level was determined by the 5,5′-dithio-bis-(2-nitrobenzoic acid) (DTNB) method [26]. In detail, 1.6 × 10^5^ HaCaT cells in 1.6 mL of DMEM were plated in each well of 6-well plates (Costar, Corning). After 12 h of incubation, the medium was removed, and 1.5 mL of DMEM containing 0.18 μg cm^−2^ Au NSs or NRs was added to each well (triplicate for each NM) for 24 h of incubation. Then, the cells were washed with PBS three times and re-suspended in cell lysis buffer on an ice bath for 5 min. An amount of 30 μL of lysate was mixed with 150 μL of 30 μg mL^−1^ DTNB in one well of a 96-well plate for 30 min of incubation. The absorbance of the resulting mixture at 412 nm was measured by a microplate reader (Varioskan Flash, Thermo Scientific).

*Mitochondrial dysfunction assessments:* Mitochondrial membrane potential and mitochondrial superoxide generation were evaluated by JC-1 (Thermo Scientific) and MitoSox Red (Thermo Scientific) fluorescent dyes, respectively [25]. Briefly, 8 × 10^4^ HaCaT cells in 800 μL of DMEM were seeded in each well of 12-well plates (Corning, Fisher Scientific) for 12 h of incubation. Then, the medium was removed, and the cells were treated with 800 μL of DMEM containing 0.18 μg cm^−2^ Au NSs or NRs for 24 h. The 12.5 μg mL^−1^ ZnO nanoparticles were used as the positive control. Then, the medium was removed, and the cells were washed with PBS three times. Subsequently, the cells were stained by 5 μmol L^−1^ JC-1 or MitoSox Red in DMEM for 30 min. Each well was gently washed with PBS three times. Finally, fluorescence imaging was acquired by an Axio Observer 7 system (ZEISS, Oberkochen, Germany).

### 2.9. Animals and Treatment 

All animal studies were performed at the SPF Animals Center, Shenzhen Institute for Drug Control, and all procedures involving experimental animals complied with the protocol approved by the Committee for Animal Research of Shenzhen Institute for Drug Control (SYXK [GD]2021-0253), China. Female NIH mice (8 weeks old, 25 ± 1 g) were purchased from the Guangdong Medical Laboratory Animal Center, quarantined, and acclimatized to laboratory conditions for 10 days prior to the experiment. 

Mice were randomly divided into 14 groups (three mice per group): control (PBS), Au NS1, Au NS2, Au NS3, NR1, NR2, NR3, control (Cream), Au NS1-Cream, Au NS2-Cream, Au NS3-Cream, Au NR1-Cream, Au NR2-Cream, and Au NR3-Cream. Prior to treatment, the backs of mice were shaved to create a 2 cm × 1 cm area, and various Au NMs (6.1 ng cm^−2^) or Au-Cream (3.3 mg cm^−2^) were dripped onto the exposed skin through uniform spreading. After 8 h of treatment, the treated skin area was gently washed with lukewarm water and dried with medical cotton. Mice were treated once a day for 30 consecutive days. At the end of treatment, mice were anesthetized intraperitoneally with a xylazine (50 mg kg^−1^)/ketamine (50 mg kg^−1^) solution, and their blood was collected by eye removal method for further blood routine examination. Then, mice were sacrificed, and the treated dorsal skin and major organs (liver, spleen, kidneys, lung, and heart) were retrieved and sectioned for hematoxylin and eosin (H and E) staining. 

Then, 40 mg of the treated skin was washed with ice-cold PBS three times and fully homogenized in an ice bath. After 10 min of centrifugation at 12,000 rpm, 120 μL of supernatant was taken for superoxide dismutase (SOD) and malondialdehyde (MDA) analysis by using a standard reagent kit (Beyotime, Shanghai, China).

### 2.10. Data Process and Analysis 

All data were given as mean ± SD based on at least triplicate experiments. One-way ANOVA was used to perform the multi-sample analysis. The significant difference level was set at *p* < 0.05.

## 3. Results 

### 3.1. Separation and Characterization of Au NMs in Commercial Cosmetics

**Figure 1:**
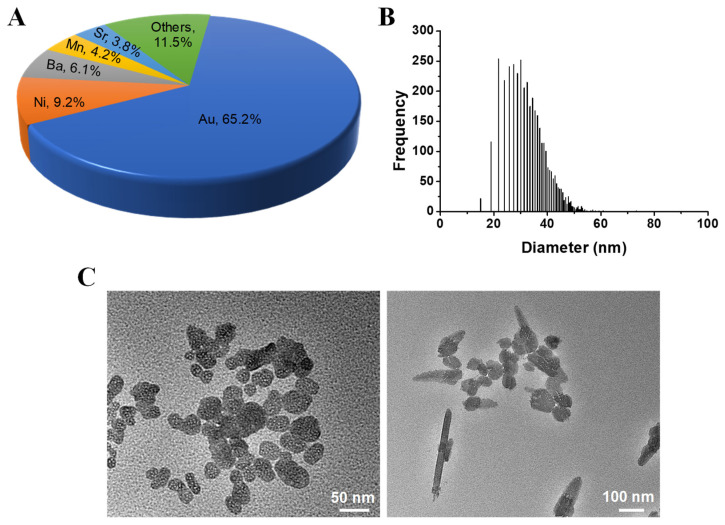
Analysis of commercial facial cream that contains Au NMs. (**A**) Metal composition analyzed by ICP−MS; (**B**) distribution of Au NMs; (**C**) TEM image of Au NMs.

One commercial facial cream from an international brand that claims to contain Au NMs was chosen to first analyze its metal content through ICP-MS. A total of 36 metal elements were involved in this initial assessment. The result revealed that the total metal content was 2.81 mg kg^−1^. Among various metal elements, Au presented the highest content of 1.83 mg kg^−1^, followed by nickel (0.26 mg kg^−1^), barium (0.17 mg kg^−1^), manganese (0.12 mg kg^−1^), and strontium (0.11 mg kg^−1^) (Figure 1A). Single-particle ICP-MS was further implemented to detect the size range of Au NMs according to the previously described method [27]. Figure 1B reveals that there exists Au NMs (Figure 1B) ranging from 15.3 to 59.4 nm. Au NMs were extracted from the cream through the sequential solvent extraction approach [20]. The TEM image showed that the Au extracts from facial cream contained various morphologies, such as spheres and rods (Figure 1C). Nanomaterials with different morphologies potentially can present different toxicity profiles. Although Au NMs could be successfully extracted and characterized, the amount of extract was not enough for a systemic toxicity evaluation. Therefore, it will be necessary to prepare the corresponding Au NMs with similar size and morphology for further toxicity assessment. 

### 3.2. Synthesis and Characterization of Au NSs and NRs

**Figure 2:**
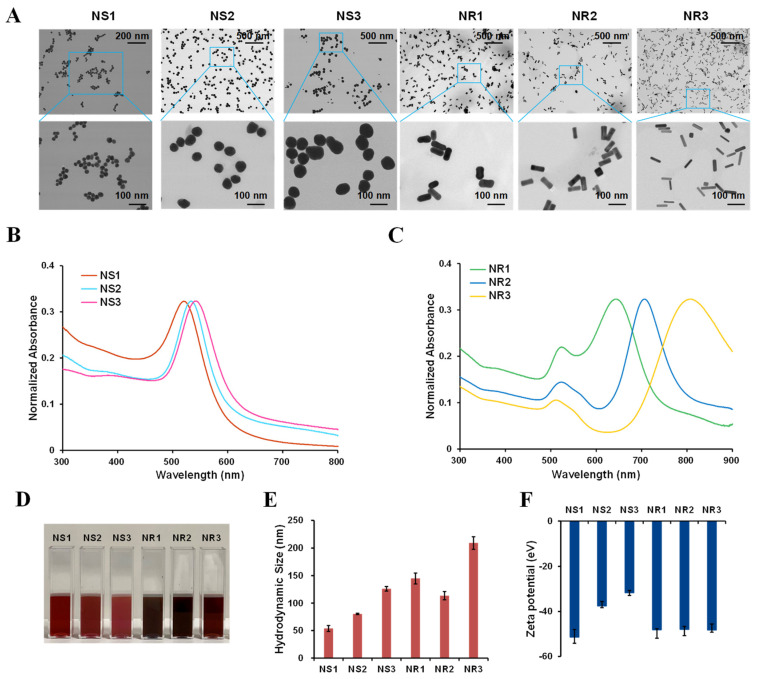
Characterization of synthesized Au NMs. (**A**) TEM images; (**B**) UV−Vis−NIR spectrum of Au NSs; (**C**) UV−Vis−NIR spectrum of Au NRs; (**D**) photographs of aqueous solution of Au NMs; (**E**) hydrodynamic size; (**F**) zeta potential.

Given that there exists spherical and rodlike Au NMs in the cream, three Au NSs with different sizes and three Au NRs with different aspect ratios were prepared. Au NSs were synthesized through citrate-reduced seeded growth methods [22]. Different sized Au NSs were achieved through adjusting the injection times of HAuCl_4_ and sodium citrate [22]. Au NRs were synthesized through CTAB-mediated seeded growth methods [23]. The length and radius of Au NRs were varied by adjusting the growth solutions containing different components, such as Au seed, AgNO_3_, H_2_SO_4_, and CuCl_2_. For further biological application, the CTAB surfactant on Au NRs was replaced by citrate through two-step displacement (first step, CTAB was replaced by PSS; second step, PSS was replaced by sodium citrate) [28]. As shown in Figure 2A, Au NSs showed spherical morphology with primary sizes of 28.1 ± 3.5 (Au NS1), 49.1 ± 4.4 (Au NS2), and 73.6 ± 5.3 nm (Au NS3), and Au NRs exhibited a rodlike shape with a length × width of (79.3 ± 9.4) nm × (40.6 ± 3.8) (Au NR1), (70.8 ± 7.7) nm × (26.1 ± 4.3) (Au NR2), and (60.0 ± 7.9) nm × (16.1 ± 2.6) nm (Au NR3), showing aspect ratios of 1.9, 2.8, and 3.8, respectively. UV-visible absorption spectra showed that Au NS1-3 had the localized surface plasmon resonance (LSPR) maximum absorption peaks at 522, 533, and 544 nm (Figure 2B), respectively, while Au NR1-3 had a similar transverse LSPR peak at 520 nm but different longitudinal LSPR peaks at 644, 707, and 808 nm (Figure 2C), respectively. All these Au NSs and NRs had color evolution, as shown in Figure 2D. In addition, based on dynamic light scattering measurements, NS1-3 showed proportionally increased hydrodynamic sizes, while those sizes of NR1-3 varied in a different manner (Figure 2E). Zeta potential measurement further revealed that all these Au NSs and NRs showed negatively charged surfaces (Figure 2F). 

### 3.3. Physicochemical Property Evaluation of Au NSs and NRs before and after Extraction from Cream

**Figure 3:**
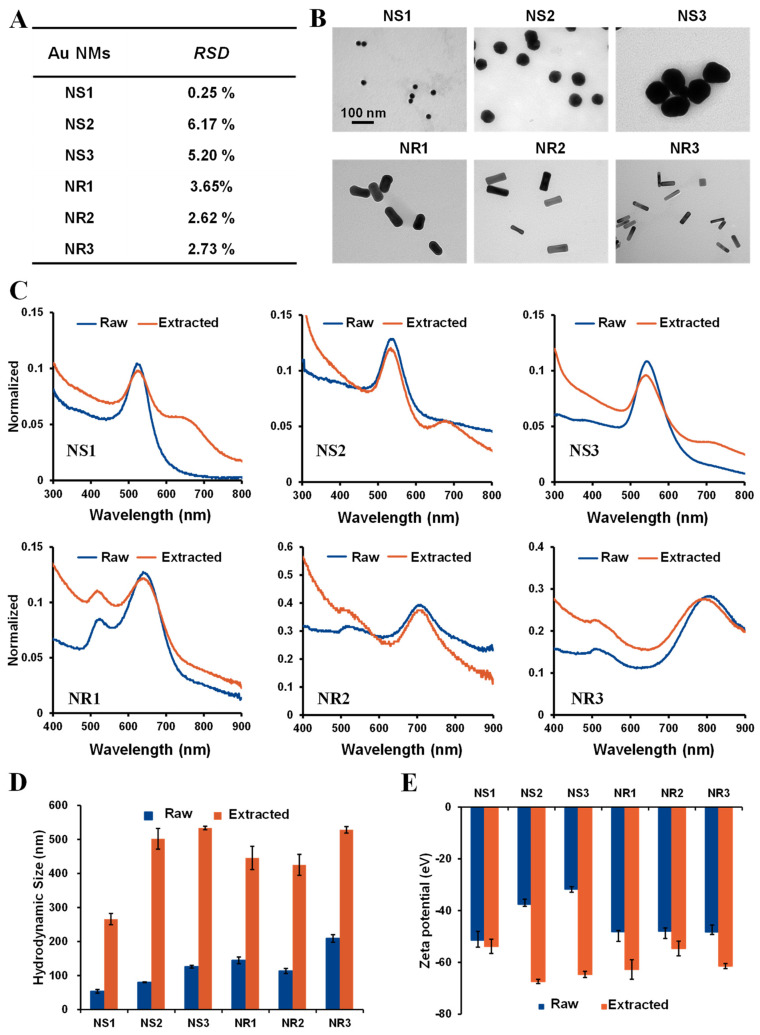
Physicochemical property comparison of Au NMs before and after extraction. (**A**) Homogeneity assessment by RSD; (**B**) TEM images of extracted Au NMs; (**C**) comparison of UV−Vis−NIR spectrum of raw and extracted Au NMs; (**D**) comparison of the hydrodynamic size of raw and extracted Au NMs; (**E**) comparison of the zeta potential of raw and extracted Au NMs.

The synthesized Au NSs and NRs were individually embedded into a commercial cream through in situ hot mixing methods. To evaluate the homogeneity of the suspension, different aliquots were taken from the suspended cream after 24 h of suspension for measurement of Au content by ICP-MS, and the relative standard deviation (RSD%) of Au content was calculated. As shown in Figure 3A, the RSD values for Au NS1-, NS2-, NS3-, NR1-, NR2-, and NR3-embedded cream were 0.25, 6.17, 5.20, 3.65, 2.62, and 2.73%, respectively, indicating the small difference and excellent homogeneity. To evaluate the potential influence of cream on the physicochemical properties of Au NSs and NRs, the sequential solvent extraction method was further applied to extract Au NSs and NRs from the cream after 24 h of stable embedment. The physicochemical properties of the extracted Au NSs and NRs were further systematically characterized. As shown in TEM images (Figure 3B), the primary size and morphology of the extracted Au NSs and NRs were not significantly different from those of the raw ones. UV-Visible absorption spectra (Figure 3C) also indicated that the LSPR peaks of various extracted Au NSs and NRs were still located at their original position, suggesting that the cream does not affect the plasmon properties of these Au NMs. However, compared with raw Au NMs, all the hydrodynamic sizes of extracted Au NMs were enlarged (Figure 3D), and their charges were altered more negatively (Figure 3E). These results mean the cream can dramatically influence the surface of Au NMs. 

### 3.4. Cellular Assessment of Toxicological Responses of Au NMs and Au NM-Cream

**Figure 4:**
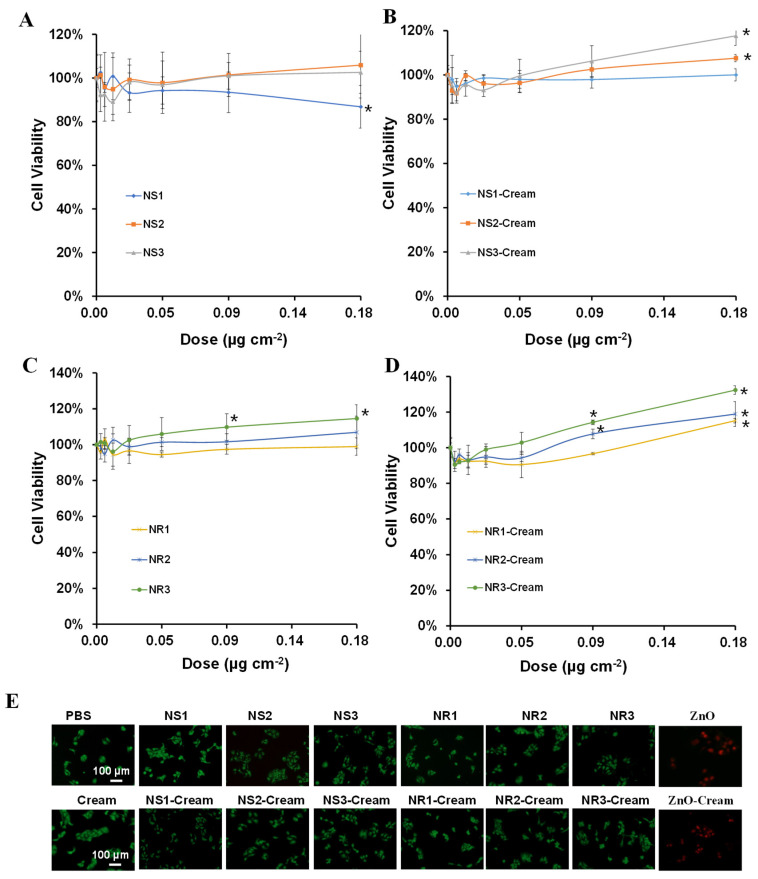
Cell viability assessment of Au NMs and Au NM−Cream in HaCaT cells. CCK-8−based viability assays of Au NS1-3 (**A**), Au NS1-3−Cream (**B**), Au NR1-3 (**C**), and Au NR1-3−Cream (**D**); * *p* < 0.05 compared with control; (**E**) fluorescence images of live/dead cells stained with Calcein AM/PI. For (**A**–**D**), HaCaT cells were treated with various concentrations of Au NMs and Au NM−Cream (equivalent to Au surface area doses) for 24 h, and the viability was assessed by CCK-8 assay. For (**E**), cells were treated with 0.18 μg cm^−2^ Au NMs and Au NM−Cream (equivalent to Au surface area doses) for 24 h, and ZnO or ZnO−Cream was used as the positive control.

**Figure 5:**
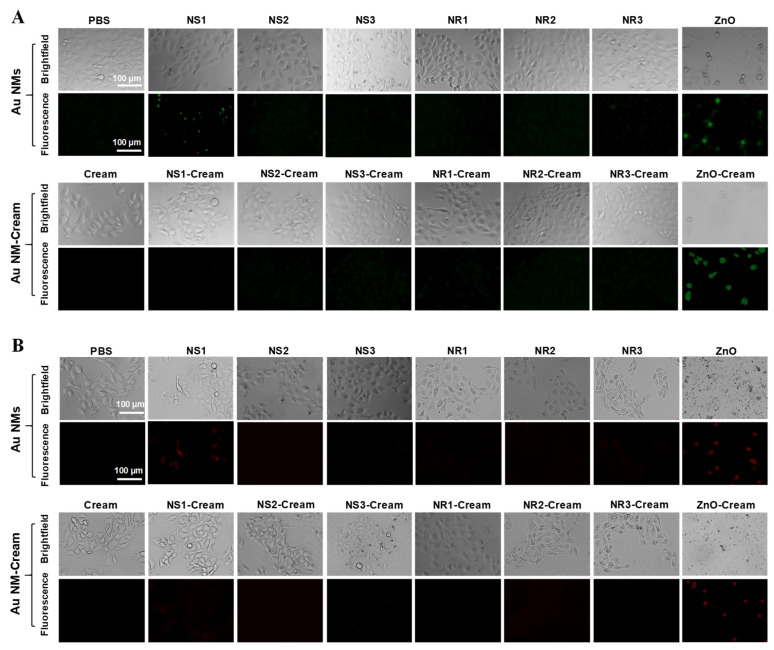
Fluorescence images to evaluate mitochondrial dysfunction induced by Au NMs and Au NM−Cream. (**A**) Mitochondrial membrane potential evaluated by JC-1; (**B**) mitochondrial superoxide generation evaluated by MitoSox Red. HaCaT cells were treated with 0.18 μg cm^−2^ Au NMs and Au NM−Cream (equivalent to Au surface area doses) for 24 h, and ZnO or ZnO−Cream was used as the positive control.

The in vitro safety of Au NMs and the Au NM-Cream was evaluated in HaCaT cells by CCK-8 assay. For Au NSs and Au NS-Cream, it was found that only Au NS1 could weakly reduce the cell viability at the highest dosage, and Au NS2 and NS3 could not affect the cell viability (Figure 4A), but Au NS1-3-Cream even weakly promoted the cell proliferation viability (Figure 4B). For Au NRs and Au NR-Cream, all the Au NR1-3 could not significantly affect the cell viability profiles (Figure 4C), while Au NR1-3-Cream significantly promoted cell proliferation (Figure 4D). GSH depletion is usually used to assess the acute oxidative injury of NMs. Cellular GSH level was evaluated by the DTNB assay. Supporting information Figure S1 shows that only Au NS1 could trigger obvious GSH depletion, while Au NS2-3, Au NS1-3-Cream, Au NR1-3, and Au NR1-3-Cream showed less effect on GSH levels. Mitochondria dysfunction was further assessed by measurements of mitochondrial membrane potential and mitochondrial superoxide levels using JC-1 and MitoSox Red fluorescence indicators. Figure 5A shows only Au NS1 induced cells to exhibit green JC-1 fluorescence and Figure 5B shows red MitoSox Red fluorescence to some extent, suggesting their potential mitochondrial depolarization and mitochondrial superoxide generation effects, while other Au NMs and Au NM-Cream did not cause any mitochondrial dysfunctions. At the same time, zinc oxide (ZnO) nanoparticles as the positive control caused both severe toxicological responses in cells. 

### 3.5. Dermal Safety Assessment of Au NMs and Au NM-Cream 

**Figure 6:**
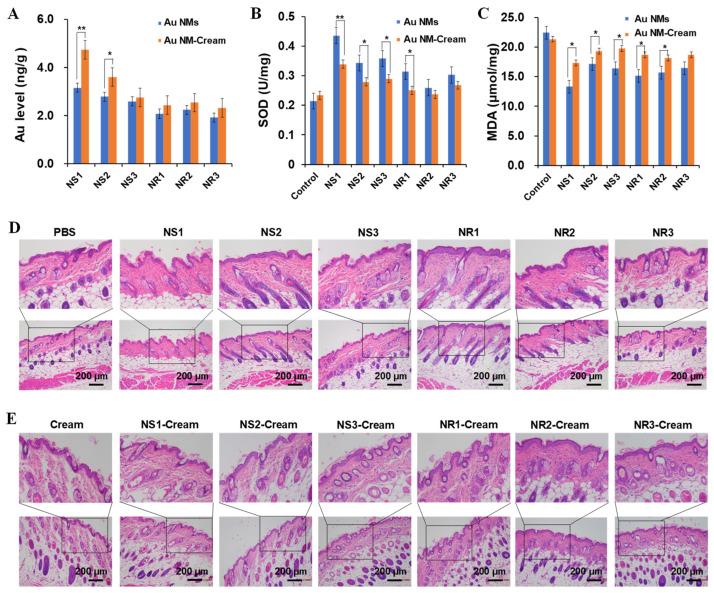
In vivo toxicological assessment of Au NMs and Au NM−Cream in hairless mice. (**A**) Au accumulation in skin; (**B**) SOD activity; (**C**) MDA content; (**D**,**E**) H and E staining of skin tissue section of mice exposed to Au NMs (**D**) and Au NM−Cream (**E**). Skin of hairless mice (*n* = 3) was daily treated with Au NMs or Au−Cream for 8 h and then gently washed with lukewarm water and dried by medical cotton. Mice were treated once a day for 30 consecutive days. Significant difference * *p* < 0.05, ** *p* < 0.01.

**Figure 7:**
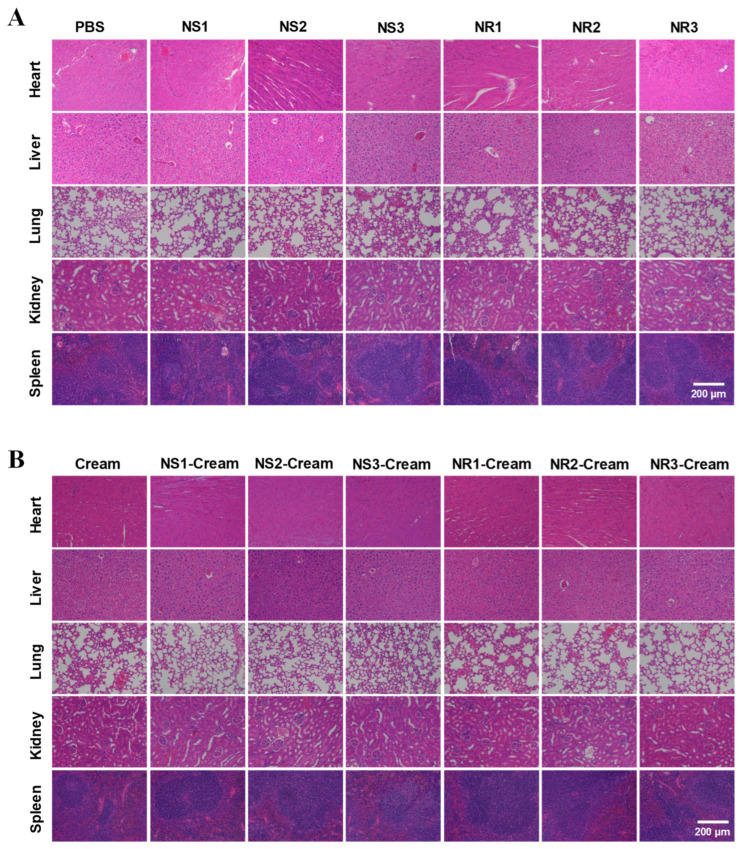
H and E staining of major organs (heart, liver, lung, kidney, spleen) of hairless mice after dermal exposure to Au NMs or Au NM−Cream for 30 days. (**A**) Au NMs; (**B**) Au NM−Cream.

In vivo dermal safety of various Au NMs and Au NM-Cream was evaluated on hairless mice models. Au NMs and Au NM-Cream were spread on the hair-shaved dorsal skin of NIH mice, and after 8 h of treatment every day, the spread area was washed with lukewarm water and dried with medical cotton. After a consecutive 30 days of treatment, skin tissue and major organs (heart, liver, lung, kidney, and spleen) were harvested to investigate the Au biodistribution by ICP-MS analysis. Au NS1-3 showed a size-dependent accumulation in skin tissues (Figure 6A), meaning the smaller Au NSs can more efficiently penetrate the skin surface. Once embedded in cream, Au NS1-3-Cream showed more accumulation in the skin, suggesting the potential promotion effect of cream in skin penetration of Au NSs. Similar results were also achieved in Au NRs and Au NR-Cream, but their accumulation levels were lower than those of Au NSs and Au NS-Cream. The significant accumulation of Au in the skin means that Au NMs can potentially penetrate the skin layer, enter the blood stream, and reach major organs, leading to systemic toxicity. However, an Au content could not be detected in major organs. Furthermore, the accumulation of Au in the skin also means that Au NMs will have sufficient opportunity to interact with skin cells and tissues, leading to potential dermal toxicity. The harvested skin tissues were further employed for the evaluation of SOD activity and MDA content. In vivo SOD activity and MDA content are indicators of antioxidant defense, and increased SOD activity and decreased MDA content indicate the oxidative stress injury. Figure 6B,C shows that all these Au NMs and the Au NM-Cream could induce increased SOD activities and decreased MDA contents, indicating weak oxidative injury, where the Au NM-Cream presented weaker responses than the Au NMs, meaning cream probably can alleviate the injury induced by Au NMs. The smallest-sized Au NS1 still induced the most potent SOD and MDA toxicological responses. However, this oxidative injury did not show any dependent manner on the size of sphere or aspect ratio of rods. Furthermore, the histological structure of skin tissues was further examined by H and E staining. Figure 6D,E shows that the thicknesses of the epidermis and dermis were not significantly altered by these Au NMs and the Au NM-Cream compared to the control (PBS) and control (Cream), respectively. However, the quantities of hair follicles and bulbs were obviously reduced by Au NSs and NRs, while they were rarely affected by the Au NS-Cream and Au NR-Cream. This result reveals the more potential risk of Au NMs compared with Au NM-Cream. Besides skin, the influence of Au NMs and Au NM-Cream on major organs (heart, liver, spleen, lung, and kidney) was also evaluated through H and E staining. As shown in Figure 7A,B, there was also no physiological or pathological changes on the major organ tissues after treatment with Au NMS and Au NM-Cream, which is consistent with the accumulation result that the Au content was undetectable in major organs. Moreover, Supporting Information Table S1 shows all the blood parameters of mice treated with Au NMs and Au NM-Cream kept at normal levels. All of these in vivo toxicological studies demonstrate that the embedment of Au NMs in cream can alleviate the toxicological potential of Au NMs.

## 4. Discussion

Au NMs have been widely used in a variety of cosmetics such as cream, lotion, face pack, deodorant, etc.; however, the safety of Au NMs in cosmetics is rarely studied. In the EU, although SCCS has safety concern about Au NMs used in cosmetics, it has not drawn any conclusion yet due to insufficient essential information and several major data gaps [29]. In the present study, a commercial facial cream was characterized to contain spherical and rodlike Au NMs; however, the quantity of Au NMs was not enough for the systematic toxicity investigation, and the individual types of Au NMs could not be purified for clarification of their toxicity profiles. Based on this difficulty, various citrate-coated Au NSs and Au NRs were prepared and further embedded into and extracted from cosmetics, affording sufficient extracted Au NMs for deep understanding of the changes in the physicochemical property. The primary sizes, morphologies, and optical properties of extracted Au NMs were found to be similar to those of raw Au NMs; however, the hydrodynamic sizes of extracted Au NMs were significantly enlarged, and their zeta potentials were changed to more negative. For cytotoxicity assessments, Au NS1 showed weak cytotoxicity against HaCaT cells at the highest dosage, while Au NS1-Cream could reduce the toxicity. Au NS2-3 and Au NR1-3 showed little cytotoxicity, while Au NS2-3-Cream and Au NR1-3-Cream could weakly and significantly promote the cell proliferation, respectively. For the dermal safety evaluation in hairless mice, all these Au NSs and NRs were found to be able to accumulate in skin tissues after 30 days of treatment, and Au NS-Cream and Au NR-Cream could further enhance this accumulation; however, there was no Au accumulation in major organs, meaning that these Au NSs and NRs cannot enter the bloodstream and be transported to the major organs. These Au NMs and the Au NM-Cream showed little alteration in the epidermis and dermis of hairless mice, but they caused SOD elevation and MDA reduction to some extent, where Au NM-Cream showed a weaker response than the Au NMs, further demonstrating the toxicity alleviation effect of cream on Au NMs. 

Citrated-coated Au NSs usually show excellent in vitro and in vivo biocompatibility and have been widely used in consumer products. The previous study has also demonstrated that citrate-coated Au NSs with sizes of 20, 50, and 70 nm are non-toxic to HaCaT keratinocytes [30]. However, there also exists the contradictory result that citrate-coated Au NSs with a size of 34 nm show mild toxicity to keratinocytes [31]. Further studies revealed that the mild toxicity of citrate-coated Au NSs should be ascribed to the acid feature of excess citrate existing on the surface of Au NSs [32,33]. In the present study, all the Au NSs and NRs were coated with citrate, and Au NS1, with the smallest size (28.1 nm), could cause weak GSH reduction, mitochondrial membrane depolarization, mitochondrial superoxide generation, and cell death in HaCaT keratinocytes at the highest dose, and it also could trigger SOD elevation and MDA reduction in the skin of hairless mice. However, these in vitro and in vivo toxicological responses were not induced by Au NS1-Cream. Similar results were also observed in Au NS2-3 and Au NR1-3 that could induce in vivo SOD elevation and MDA reduction to some extent but failed to induce them after embedment into the cream. All these results demonstrate that the complicated ingredients in the cream probably interact with Au NSs and NRs and capture the toxic citrate, resulting in toxicity removal. Previous studies have investigated the impact of cosmetic cream on the toxicity of Au nanosheets [20,21]. Cosmetic cream was found to enhance the accumulation of Au nanosheets in the skin but could not facilitate their transportation to the bloodstream and major organs, which is consistent with our results. However, cosmetic cream was found to strengthen the cytotoxicity of Au nanosheets, probably due to the interaction of Au nanosheets with the ingredients of cosmetic cream, which is different from our findings. Different ingredients of cosmetic creams from different commercial brands probably induce these differences. In the present study, the surface of raw Au NMs, once embedded into the cream, probably was covered by the ingredients of the cream through physical absorption or chemical binding modes, which resulted in enlarged hydrodynamic sizes, strengthened negative charges, or reduced toxicity. However, at this moment, we cannot clearly understand these interactions due to the lack of exact information about the ingredients of the cream. In future studies, homemade cream can be used to investigate these physicochemical features and toxicity profiles again and clarify the roles of various ingredients.

## 5. Conclusions

Embedment in cream can obviously affect hydrodynamic size and zeta potentials on Au NSs and NRs but has little influence on their primary sizes, morphologies, and optical properties. Au NSs with the smallest size of 28.1 nm induced weak in vitro and in vivo toxicological responses, which can be alleviated after embedment in cream. Moreover, other Au NSs with sizes of 49.1 and 73.6 nm and NRs with aspect ratios of 1.9, 2.8, and 3.8 showed non-toxicity against cells, but they triggered weak in vivo toxicological responses, which are also alleviated after embedment in cream. Based on this study, the interaction between Au NMs and ingredients of cosmetics seems to play a critical role in toxicity enhancement or reduction, and in future studies, the mode and process of this interaction at the molecular level should be clarified, which will be helpful to broaden the application of nanomaterials in cosmetics.

## Supplementary Materials

The following supporting information can be downloaded at: https://www.mdpi.com/article/10.3390/toxics10060276/s1, Figure S1: GSH level of cells induced by Au NMs and Au NM-Cream; Table S1: Blood parameters of mice after dermal exposure to Au NMs and Au NM-Cream for 30 days.
