# Supplementary material for: Dermal Toxicity Influence of Gold Nanomaterials after Embedment in Cosmetics
Source: Toxics. 2022 May 24;10(6):276. doi: 10.3390/toxics10060276 (PMC9228324; doi:10.3390/toxics10060276)
Supplement: Supplementary file 1 [file toxics-10-00276-s001.zip › toxics-1725431-supplementary.pdf]

Supplementary Materials: Dermal Toxicity Influence of Gold Nanomaterials after Embedment in Cosmetics

Chusheng Liu, Yanjing Wang, Gaofei Zhang, Xuebin Pang, Jiao Yan, Xiaoou Wu, Yingheng Qiu, Ping Wang, Houshuang Huang, Xiaowei Wang and Haiyuan Zhang

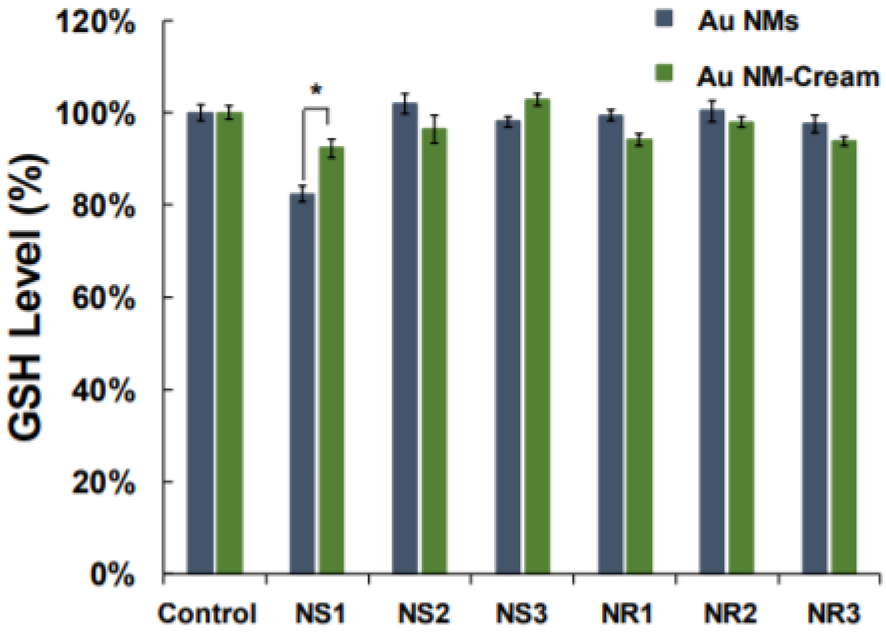

Figure S1. GSH level of cells induced by Au NMs and Au NM-Cream. HaCaT cells were treated with 0.18 μg cm-2 Au NMs and Au NM-Cream (equivalent to Au surface area doses) for 24 h, and GSH level was assessed by DTNB methods. Significant difference *p<0.05.

Table S1. Blood parameters of mice after dermal exposure to Au NMs and Au NM-Cream for 30 days.

	PBS	NS1	NS2	NS3	NR1	NR2	NR3
WBC(10^9/L)	4.3 ± 1.52	2.85 ± 0.3	3.32 ± 1.08	4.4 ± 0.78	4.37 ± 0.68	3.97 ± 0.41	5.06 ± 1.74
RBC(10^12/L)	10.28 ± 0.18	10.77 ± 1.1	10.01 ± 0.26	10.15 ± 0.55	10.24 ± 0.24	10.25 ± 0.38	10.62 ± 0.27
HGB(g/L)	163 ± 7.55	171 ± 8.49	155.67 ± 3.06	162 ± 4.58	160.67 ± 4.04	160.33 ± 5.51	169 ± 1.73
PLT(10^9/L)	1084.67 ± 122.32	1274.5 ± 320.32	1114.67 ± 98.19	1260.33 ± 19.4	1101.67 ± 55.77	811.33 ± 116.37	1155 ± 137.36
NEUT%(%)	17.9 ± 3.03	12.5 ± 5.23	14.2 ± 0.5	65.03 ± 44.23	87.87 ± 5.99	86.93 ± 6.06	65.13 ± 45.36
LYMPH%(%)	77.17 ± 3.11	82.65 ± 2.76	79.4 ± 5.28	31.37 ± 46.05	9.1 ± 6.02	9.7 ± 6.01	31.37 ± 45.32
	**Cream**	**NS1-Cream**	**NS2-Cream**	**NS3-Cream**	**NR1-Cream**	**NR2-Cream**	**NR3-Cream**
WBC(10^9/L)	3.53 ± 0.95	5.32 ± 1.64	4.78 ± 1.44	4.86 ± 0.62	3.51 ± 0.76	4.05 ± 0.4	3.83 ± 0.57
RBC(10^12/L)	10.41 ± 0.15	10.02 ± 0.61	10.28 ± 0.24	9.67 ± 0.67	10.37 ± 0.69	10.21 ± 0.31	10.2 ± 0.21
HGB(g/L)	161.67 ± 2.08	163.33 ± 1.53	151.67 ± 2.52	153 ± 5.2	156.67 ± 4.73	154.67 ± 9.07	155 ± 4.58
PLT(10^9/L)	1201 ± 113.41	994.33 ± 105.31	1244 ± 135.6	1165.33 ± 108.03	1185.33 ± 54.86	1085 ± 42.23	1261.67 ± 177.4
NEUT%(%)	9.27 ± 4.37	8.03 ± 3.18	6.17 ± 1.89	4.8 ± 1.41	10.1 ± 3.86	9.07 ± 1.62	9.13 ± 3.94
LYMPH%(%)	81.87 ± 5.52	86.6 ± 4.22	87.9 ± 2.3	88.63 ± 4.74	82.9 ± 3.99	85.8 ± 2.77	83.17 ± 4.42
